# Prevalence of Cannabis Withdrawal Symptoms Among People With Regular or Dependent Use of Cannabinoids: A Systematic Review and Meta-analysis

**DOI:** 10.1001/jamanetworkopen.2020.2370

**Published:** 2020-04-09

**Authors:** Anees Bahji, Callum Stephenson, Richard Tyo, Emily R. Hawken, Dallas P. Seitz

**Affiliations:** 1 Department of Public Health Sciences, Queen’s University, Kingston, Ontario, Canada; 2 Department of Psychiatry, Queen’s University, Kingston, Ontario, Canada; 3 Queen’s University School of Kinesiology and Health Studies, Kingston, Ontario, Canada; 4 Cumming School of Medicine, Department of Psychiatry, University of Calgary, Calgary, Alberta, Canada

## Abstract

**Importance:**

Cannabis withdrawal syndrome (CWS)—a diagnostic indicator of cannabis use disorder—commonly occurs on cessation of heavy and prolonged cannabis use. To date, the prevalence of CWS syndrome has not been well described, nor have the factors potentially associated with CWS.

**Objectives:**

To estimate the prevalence of CWS among individuals with regular or dependent use of cannabinoids and identify factors associated with CWS.

**Data Sources:**

A search of literature from database inception to June 19, 2019, was performed using MEDLINE, Embase, PsycINFO, Web of Science, the Cumulative Index to Nursing and Allied Health Literature, ProQuest, Allied and Complementary Medicine, and Psychiatry online, supplemented by manual searches of reference lists of included articles.

**Study Selection:**

Articles were included if they (1) were published in English, (2) reported on individuals with regular use of cannabinoids or cannabis use disorder as a primary study group, (3) reported on the prevalence of CWS or CWS symptoms using a validated instrument, (4) reported the prevalence of CWS, and (5) used an observational study design (eg, cohort or cross-sectional).

**Data Extraction and Synthesis:**

All abstracts, full-text articles, and other sources were reviewed, with data extracted in duplicate. Cannabis withdrawal syndrome prevalence was estimated using a random-effects meta-analysis model, alongside stratification and meta-regression to characterize heterogeneity.

**Main Outcomes and Measures:**

Cannabis withdrawal syndrome prevalence was reported as a percentage with 95% CIs.

**Results:**

Of 3848 unique abstracts, 86 were selected for full-text review, and 47 studies, representing 23 518 participants, met all inclusion criteria. Of 23 518 participants included in the analysis, 16 839 were white (72%) and 14 387 were men (69%); median (SD) age was 29.9 (9.0) years. The overall pooled prevalence of CWS was 47% (6469 of 23 518) (95% CI, 41%-52%), with significant heterogeneity between estimates (*I*^2^ = 99.2%). When stratified by source, the prevalence of CWS was 17% (95% CI, 13%-21%) in population-based samples, 54% in outpatient samples (95% CI, 48%-59%), and 87% in inpatient samples (95% CI, 79%-94%), which were significantly different (*P* < .001). Concurrent cannabis (β = 0.005, *P* < .001), tobacco (β = 0.002, *P* = .02), and other substance use disorders (β = 0.003, *P* = .05) were associated with a higher CWS prevalence, as was daily cannabis use (β = 0.004, *P* < .001).

**Conclusions and Relevance:**

These findings suggest that cannabis withdrawal syndrome appears to be prevalent among regular users of cannabis. Clinicians should be aware of the prevalence of CWS in order to counsel patients and support individuals who are reducing their use of cannabis.

## Key Points

**Questions:**

What is the prevalence of cannabis withdrawal syndrome among individuals with regular or dependent use of cannabis, and which factors are associated with cannabis withdrawal syndrome?

**Findings:**

In this meta-analysis of observational studies including 23 518 participants, the prevalence of cannabis withdrawal syndrome was found to be 47%. Factors that were associated with higher cannabis withdrawal syndrome were clinical settings (particularly inpatient and outpatient vs population settings), concurrent tobacco or other substance use, and daily cannabis use.

**Meaning:**

Cannabis withdrawal syndrome appears to be common among regular users of cannabis, particularly those in outpatient and inpatient settings and individuals with substance use disorders; clinicians should be aware of the high prevalence of cannabis withdrawal syndrome to counsel patients and support individuals who are reducing their use of cannabis.

## Teaser

This systematic review and meta-analysis examines the prevalence of cannabis withdrawal syndrome in individuals who use cannabinoids regularly.

## Introduction

Cannabinoids are the most commonly used group of illicit drugs, and cannabis use and dependence are estimated to have increased over the past 2 decades.^1^ Despite common perceptions that cannabis is relatively harmless, there is substantial evidence to support an association between cannabis use and several medical, neurocognitive, functional, and psychosocial sequalae.^2^ The known short-term risks of cannabinoid use include impaired short-term memory and motor coordination, altered judgment, paranoia, and psychosis.^3^ Similarly, long-term effects of cannabinoid use include addiction, altered brain development, poor educational outcomes, cognitive impairment, diminished quality of life, increased risk of chronic respiratory tract and psychotic disorders, injuries, motor vehicle collisions, and suicide.^3,4^

In parallel with other substance withdrawal syndromes, a cannabis withdrawal syndrome (CWS)—originally proposed by Budney and colleagues^5,6,7,8^ —has received recognition in recent years. Cannabis withdrawal syndrome symptoms occur reliably following a specific time course with cessation of cannabis use, were transient, could be ameliorated by readministration of cannabis, and were clinically significant. Cannabis withdrawal syndrome was recognized by the *Diagnostic and Statistical Manual of Mental Disorders, Fifth Edition*,^9^ and requires the presence of at least 3 of the following symptoms developing within 7 days of reduced cannabis use: (1) irritability, anger, or aggression; (2) nervousness or anxiety; (3) sleep disturbance; (4) appetite or weight disturbance; (5) restlessness; (6) depressed mood; and (7) somatic symptoms, such as headaches, sweating, nausea, vomiting, or abdominal pain.

Several studies using varied approaches have characterized CWS, and resulting prevalence estimates have ranged from 11.1% to 94.2%.^8,10,11,12^ Hence, although there is concern about the risks associated with cannabinoid use and CWS, to our knowledge, there currently exists no comprehensive quantitative synthesis of the magnitude of risk and how elevated that risk might be relative to the general population among people with regular or problematic cannabinoid use.

The primary aim of this systematic review and meta-analysis was to estimate the prevalence of CWS and identify contributors to heterogeneity in reported results. We sought to produce age-specific and sex estimates of CWS prevalence where possible.

## Methods

Using an a priori protocol,^13^ we conducted our systematic review in accordance with the Preferred Reporting Items for Systematic Reviews and Meta-analyses (PRISMA) reporting guideline.^14^ The need for institutional review board approval was waived by Queen’s University because this systematic review does not constitute human subject research. The search strategy was developed in consultation with a research librarian. Eight electronic databases (MEDLINE, Embase, PsycInfo, Web of Science, Allied and Complementary Medicine, Cumulative Index to Nursing and Allied Health Literature, ProQuest, and Psychiatry Online) were searched from inception to June 19, 2019, with no restriction on the year of the study. Medical Subject Headings and key words related to cannabis withdrawal, cannabis use, and prevalence of epidemiologic factors were used (eTable 1 in the Supplement). The reference lists of all included full-text articles were searched to identify any studies missed in the initial search, and the PubMed similar articles feature was used to find additional academic articles citing eligible articles. References that consisted of abstracts alone were not considered. References were compiled and managed using Zotero (George Mason University).^15^ Citations were then imported into the web-based screening tool Covidence (Cochrane Collaboration),^16^ where duplicate citations were removed.

Titles and abstracts were screened by one reviewer (A.B.), and all material marked as excluded was reviewed by a second person (R.T.) to ensure accuracy in first-pass screening. At this stage, the criteria were purposely broad to allow inclusion of any relevant studies. To be included, studies had to be published in English and report original research using any observational design (eg, cross-sectional or cohort) that reported on CWS in individuals with regular or dependent cannabis or synthetic cannabinoid use. The exact definition of regular cannabinoid use varied across cohorts, and we summarize the studies’ criteria and characteristics in Table 1.^17,18,19,20,21,22,23,24,25,26,27,28,29,30,31,32,33,34,35,36,37,38,39,40,41,42,43,44,45,46,47,48,49,50,51,52,53,54,55,56,57,58,59,60^ Case reports and series were excluded. Full-text articles were screened by 2 independent reviewers (A.B. and C.S.), with discrepancies resolved by consensus or via consultation with a third reviewer (R.T., E.R.H., or D.P.S.) when consensus was not reached. Articles were included if they (1) were published in English, (2) reported individuals with regular or dependent cannabinoid use as a primary study group, (3) reported CWS or CWS symptoms using a validated instrument, and (4) reported the prevalence of CWS in individuals with regular or dependent cannabinoid use. For studies that used the same sample of data, those providing the most detailed information were included, and the others were kept for reference.

**Table 1.** Characteristics of Included Studies

Source	Study setting	Criteria		No.	CUD, %	Male, %	Age, y	CWS, %
		CUD	CWS					
Cottler et al,^17^ 1995, United States	Population	*DSM-III-R*	CIDI-SAM	102	8	57	37.0	15.7
Wiesbeck et al,^18^ 1996, United States	Population	*DSM-IV*	SSAGA	1735	50.4	63	32.3	15.6
Budney et al,^19^ 1998, United States	Outpatient	*DSM-III-R*	Operationalized	62	100	87	31.2	75.8
Crowley et al,^20^ 1998, United States	Outpatient	*DSM-III-R*	CIDI-SAM, DISC	229	78.6	72	15.8	66.8
Swift et al,^21^ 1998, Australia	Population	*DSM-III-R*	Operationalized	243	57	58	36.0	20.2
Budney et al,^6^ 1999, United States	Outpatient	*DSM-III-R*	MWC	54	54	85	33.8	57.4
Schuckit et al,^22^ 1999, United States	Outpatient	*DSM-III-R*	Operationalized	596	30	66.1	32.0	39.9
Kouri and Pope et al,^23^ 2000, United States	Outpatient	*DSM-IV*	Self-reported diary	30	100	87	42.5	60.0
Swift et al,^24^ 2000, Australia	Outpatient	*DSM-III-R*	Operationalized	162	92	53.7	30.0	32.1
Swift et al,^25^ 2001, Australia	Population	*DSM-IV*	CIDI, *DSM-IV*, SCID	722	20.8	NA	NA	29.5
Stephens et al,^26^ 2002, United States	Outpatient	*DSM-IV*	SCID, TLFB, ASI	450	100	68.4	36.1	77.6
Budney et al,^7^ 2003, United States	Outpatient	*DSM-IV*	MWC, MCQ	18	100	61	30.9	77.8
Vandrey et al,^27^ 2005, United States	Outpatient	*DSM-IV*	MWC, YSR, WDS	72	56.9	90	16.2	58.3
Copersino et al,^28^ 2006, United States	Outpatient	*DSM-IV*	MJQQ	104	54	78	35.0	44.2
Levin et al,^29^ 2006, United States	Outpatient	*DSM-IV*	CMR, URICA, RDU	42	100	74	34.3	69.0
Nocon et al,^30^ 2006, Germany	Population	*DSM-IV*	CIDI-SAM, MWC	732	3.5	NA	19.0	16.1
Lukasiewicz et al,^31^ 2007, France	Population	*DSM-IV*	Operationalized	278	26.7	90.1	39.0	7.6
Agrawal et al,^32^ 2008, United States	Population	*DSM-IV*	AUDADIS	1603	12.2	62	30.8	8.0
Chung et al,^33^ 2008, United States	Outpatient	*DSM-IV*	MWC, SCID	214	60.7	67	16.8	36.9
Cornelius et al,^34^ 2008, United States	Outpatient	*DSM-IV*	MWC	170	100	54	20.3	43.5
Hasin et al,^35^ 2008, United States	Population	*DSM-IV*	SCID	2613	57.2	67	58.5	34.4
Jungerman et al,^36^ 2008, Brazil	Outpatient	*DSM-III-R*	CIDI, TFLB, MWC	160	100	80	32.3	51.3
Milin et al,^37^ 2008, Canada	Inpatient	*DSM-IV*	CWS, SCID	21	100	67	17.0	100.0
Vandrey et al,^38^ 2008, United States	Inpatient	*DSM-IV*	WSC	12	100	50	28.2	100.0
Mennes et al,^39^ 2009, United States^a^	Outpatient	*DSM-IV*	CIDI-SAM	416	48	49	22.0	50.0
Mennes et al,^39^ 2009, United States^a^	Outpatient	*DSM-IV*	CIDI-SAM	278	63	49	22.0	68.0
Ehlers et al,^40^ 2010, United States	Population	*DSM-IV*	SSAGA	818	13.9	38	48.4	16.5
Levin et al,^41^ 2010, United States	Outpatient	*DSM-IV*	MJQQ	469	91	58	31.2	42.4
Preuss et al,^42^ 2010, Germany	Inpatient	*DSM-IV*	MWC	118	100	85	19.6	72.0
Vorspan et al,^43^ 2010, United States^a^	Outpatient	*DSM-IV*	MJQQ	43	79.1	69.8	37.0	65.1
Vorspan et al,^43^ 2010, United States^a^	Outpatient	*DSM-IV*	MJQQ	56	100	71.4	27.0	64.3
Dervaux et al,^44^ 2011, France	Inpatient	*DSM-IV*	DIGS	92	100	75	28.7	84.8
Gorelick et al,^45^ 2012, United States	Outpatient	*DSM-IV*	MJQQ, self-report diary	384	92.4	58.3	29.2	40.9
Boggs et al,^46^ 2013, United States	Outpatient	*DSM-IV*	MJQQ	120	81.7	77	41.5	50.0
Smith et al,^47^ 2013, United States^a^	Population	*DSM-IV*	AUDADIS	1712	NA	68	34.3	18.8
Smith et al,^47^ 2013, United States^a^	Population	*DSM-IV*	AUDADIS	1187	NA	68	34.3	9.8
Verweij et al,^48^ 2013, Australia	Population	*DSM-IV*	SSAGA, CWS, MCQ	2276	23.6	39	31.9	11.9
Bonnet et al,^49^ 2014, Germany	Inpatient	*DSM-IV*	MWC	39	100	80	28.6	92.3
Greene et al,^50^ 2014, United States	Outpatient	*DSM-IV*	CDDR	90	84.4	82	16.6	40.0
Lee et al,^51^ 2014, United States	Inpatient	*DSM-IV*	CWS, MCQ, SCL-90R	30	79.3	100	28.5	73.3
Delforterie et al,^52^ 2015, United States^a^	Population	*DSM-IV*	AUDADIS, CIDI	1568	11.7	50	24.8	29.2
Delforterie et al,^52^ 2015, the Netherlands^a^	Population	*DSM-IV*	AUDADIS, CIDI	359	16.4	65	23.9	12.5
Herrmann et al,^53^ 2015, United States	Outpatient	*DSM-5*	MWC, WDS	136	77.9	73	33.3	50.7
Macfarlane and Christie,^54^ 2015, New Zealand	Inpatient	*DSM-IV*	MWC	47	100	63	31.0	87.2
Soenksen et al,^55^ 2015, United States	Outpatient	*DSM-IV*	MWC	93	76.9	100	16.4	66.7
Davis et al,^56^ 2016, United States	Outpatient	*DSM-IV*	CWS	110	53.4	93	19.2	48.2
Sherman et al,^57^ 2017, United States	Outpatient	*DSM-5*	TFLB, MWC, MCQ	302	100	72	30.3	50.3
Chauchard et al,^58^ 2018, United States	Outpatient	*DSM-IV*	MJQQ	23	100	82.6	27.4	30.4
Livne et al,^59^ 2019, United States	Population	*DSM-5*	*DSM-5*	1527	24.6	66	NA	12.1
Perron et al,^60^ 2019, United States	Outpatient	*DSM-5*	MWC	801	1.8	53.6	45.1	52.3

Abbreviations: ASI, Addiction Severity Index; AUDADIS, Alcohol Use Disorder and Associated Disabilities Schedule; CDDR, Customary Drinking and Drug Use Record; CIDI-SAM, Composite International Diagnostic Interview–Substance Abuse Module; CMR, Circumstances, Motivation, Readiness; CUD, cannabis use disorder; CWS, cannabis withdrawal syndrome; DIGS, Diagnostic Interview for Genetic Studies; DISC, Diagnostic Interview Schedule for Children; *DSM-III-R*, *Diagnostic and Statistical Manual of Mental Disorders*; *DSM-IV*, *Diagnostic and Statistical Manual, Fourth Edition*; *DSM-5*, *Diagnostic and Statistical Manual, Fifth Edition*; MCQ, Marijuana Cravings Questionnaire; MJQQ, Marijuana Quit Questionnaire; MWC, Marijuana Withdrawal Checklist; SCID, Structured Clinical Interview for *DSM*; SCL-90R, Symptom Checklist 90–Revised; SSAGA, Semi-Structured Assessment for the Genetics of Alcoholism; TFLB, time-line follow-back; WDS, Withdrawal Discomfort Scale; WSC, Withdrawal Symptom Checklist; YSR, Youth Self-Report.

^a^ These studies included 2 or more substudies.

The data extraction form was developed in Microsoft Excel 2016 (Microsoft Corp) based on previously conducted reviews^12,61,62^ and recommendations outlined in the STROBE statement (eTable 2 in the Supplement).^63^ Data were independently extracted by 1 member of the research team (A.B.) and checked by a second (C.S.). Bibliographic information was extracted in addition to study-specific data.

The following data were abstracted: study information (ie, author, journal, and year of publication), study characteristics (ie, study setting, country of study, and duration of follow-up), participant characteristics (ie, age, comorbidities, substance use, and race/ethnicity), condition information (ie, data sources, condition definition, and total number of participants), the prevalence of CWS, or the information necessary to calculate an estimate.

Data on the prevalence of CWS information were extracted and, where possible, grouped to be consistent with previous CWS rating instruments developed by cannabinoid expert groups (eTable 3 in the Supplement).^64,65^ If data reporting in the publications was incomplete, supplementary information and documents were searched to locate missing data. If supplementary information could not be located or did not provide the necessary data needed, primary study authors were contacted by email for additional information.

The quality of studies was assessed using the Newcastle-Ottawa Scale for observational studies.^66^ This scale uses a star system to evaluate nonrandomized studies regarding 3 domains of quality (selection, comparability, and outcome) using 8 criteria: representativeness of the exposed cohort, selection of the nonexposed cohort, ascertainment of exposure, demonstration that the outcome of interest was not present at the start of the study, comparability of cohorts on the basis of the design or analysis, assessment of outcome, sufficient length of follow-up for outcomes to occur, and adequacy of follow-up of the cohort. Individual star scores for each criterion were tallied to provide an overall quality score, where the greater the quality score, the higher the methodologic quality of the study (maximum score: 8 points). Studies that achieved a total rating of 6 points or higher were considered to be of the highest quality, studies that achieved a total rating of fewer than 2 points were considered to be of lowest quality, and those between 2 and 5 points were rated as fair quality. Study information necessary for quality assessment was extracted to the Excel template by one reviewer (A.B.) and double checked by a second (C.S.). Discrepancies were resolved via consultation with a third reviewer (R.T., E.R.H. or D.P.S.).

### Statistical Analysis

Descriptive statistics were calculated using proportions and means and compared using *t* tests or χ^2^ tests where appropriate. For all tests, 2-sided *P* values <.05 were considered statistically significant. Study settings included nonclinical, population-based studies, outpatient clinical studies, or inpatient clinical settings. Informant-rated scales were those completed by a family member or other informant familiar with the participant. If studies used multiple cut points to calculate CWS, the lowest threshold for defining CWS was selected.

A random-effects model for meta-analysis was used because of assumed heterogeneity between the studies. The metafor package in R, version 1.1.463 (R Studio) was used to produce the pooled estimates, forest plots, and meta-regression.^67^ The meta-analysis of proportions uses the binomial distribution to model the within-study variability or by allowing Freeman-Tukey double arcsine transformation to stabilize the variances.^68^ Heterogeneity was quantified using the *I*^2^ statistic, and its significance was determined based on the accompanying Cochran *Q* test *P* value.^69^ An *I*^2^ value of 0% indicates no observed heterogeneity, and increasing values represent greater amounts of heterogeneity; values of 25%, 50%, and 75% indicate low, moderate, and high levels of heterogeneity, respectively.^69^

Subgroup analyses were planned for accessing the associations of study population source (population or clinic based), method of CWS diagnosis (informant rated, self-report, or clinician administered), geographic location, intensity of cannabis use, sex, psychiatric comorbidity, and age with the prevalence of CWS in patients with regular or dependent use of cannabinoids. However, where studies did not report subgroup-level estimates within primary studies, we applied random-effects meta-regression to assess the association between the variable and prevalence of CWS.^70^

Publication bias was assessed qualitatively, using funnel plot symmetry as a surrogate for low risk of publication bias, as well as quantitatively, using the Egger and trim-and-fill methods.^71,72,73^ Supplementary analyses are outlined in the eFigure 1 in the Supplement.

## Results

**Figure 1.**
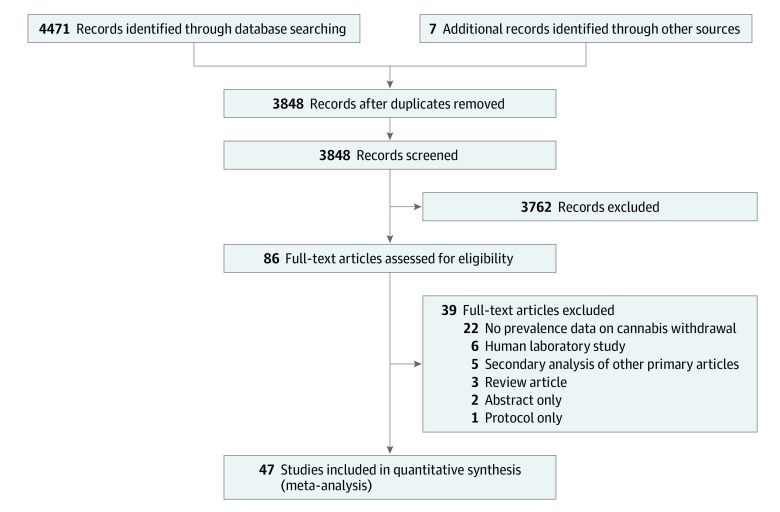
PRISMA Flowchart of Study Selection Search and selection process applied during the systematic review.

We screened a total of 3848 unique citations, of which 86 were screened in full, and 47 were included in the review (Figure 1), reporting on 50 unique cohorts. In total, 23 518 participants were represented across cohorts (median [SD] age, 29.9 [9.0] years; 16 839 white [72%]; and 14 387 men [69%]). Twenty-five cohorts (50%) were of treatment-seeking individuals. Most of the cohorts came from North America (38 [76%]), Australia (7 [14%]), or Europe (6 [12%]) (Table 1). Participants in included sources were obtained from primarily clinical samples (inpatient: 7 [14%] and outpatient: 28 [56%]) or population-based samples (15 [30%]). Individual cohorts varied widely in size (12-2613). Reporting of cohort demographics was incomplete; for example, fewer than half of the cohorts reported the baseline cannabis intake. Eighteen cohorts reported the percentage who had experienced lifetime CWS, and the remaining 32 reported current (past year) CWS prevalence.

Cannabis withdrawal syndrome was identified by a variety of clinician-administered instruments (including the Cannabis Withdrawal Scale^74^), self-reported rating scales (including the Marijuana Withdrawal Symptom Checklist^6^), and semistructured clinical interviews (involving the Time-Line-Follow-Back^75^ and the Structured Clinical Interview for the *DSM*^76^). Across studies, the specific instruments used were the Alcohol Use Disorder and Associated Disabilities Schedule,^32,47,52^ the Customary Drinking and Drug Use Record,^50^ the Composite International Diagnostic Interview-Substance Abuse Module,^17,20,25,30,36,39^ the Cannabis Withdrawal Scale,^37,51,54,56^ the Marijuana Quit Questionnaire,^28,41,43,45,46,58^ the Marijuana Withdrawal Symptom Checklist,^6,7,27,33,34,42,49,53,55,60^ the Semi-Structured Assessment for the Genetics of Alcoholism,^18,40,48^ the Structured Clinical Interview for the *DSM*,^19,21,22,23,24,26,31,35,59^ and the Time-Line-Follow-Back.^57^

Cannabis use disorder (CUD) or its equivalent (ie, cannabis dependence with or without cannabis abuse) was analyzed as defined by the study authors using varying criteria sets, including the *Diagnostic and Statistical Manual of Mental Disorders, Fifth Edition*^77^ or *International Statistical Classification of Diseases and Related Health Problems, 11th Revision*,^78^ with or without the use of interview guides, such as the Mini-International Neuropsychiatric Interview^79^ or the Structured Clinical Interview for the *DSM*.^76^ The overall proportion of participants with CUD was 34.7% (n = 8275). In population studies, estimates ranged from 8% to 34%. In outpatient-based samples, estimates ranged from 30% to 74%. In inpatient-based samples, estimates ranged from 72% to 98%.

**Figure 2.**
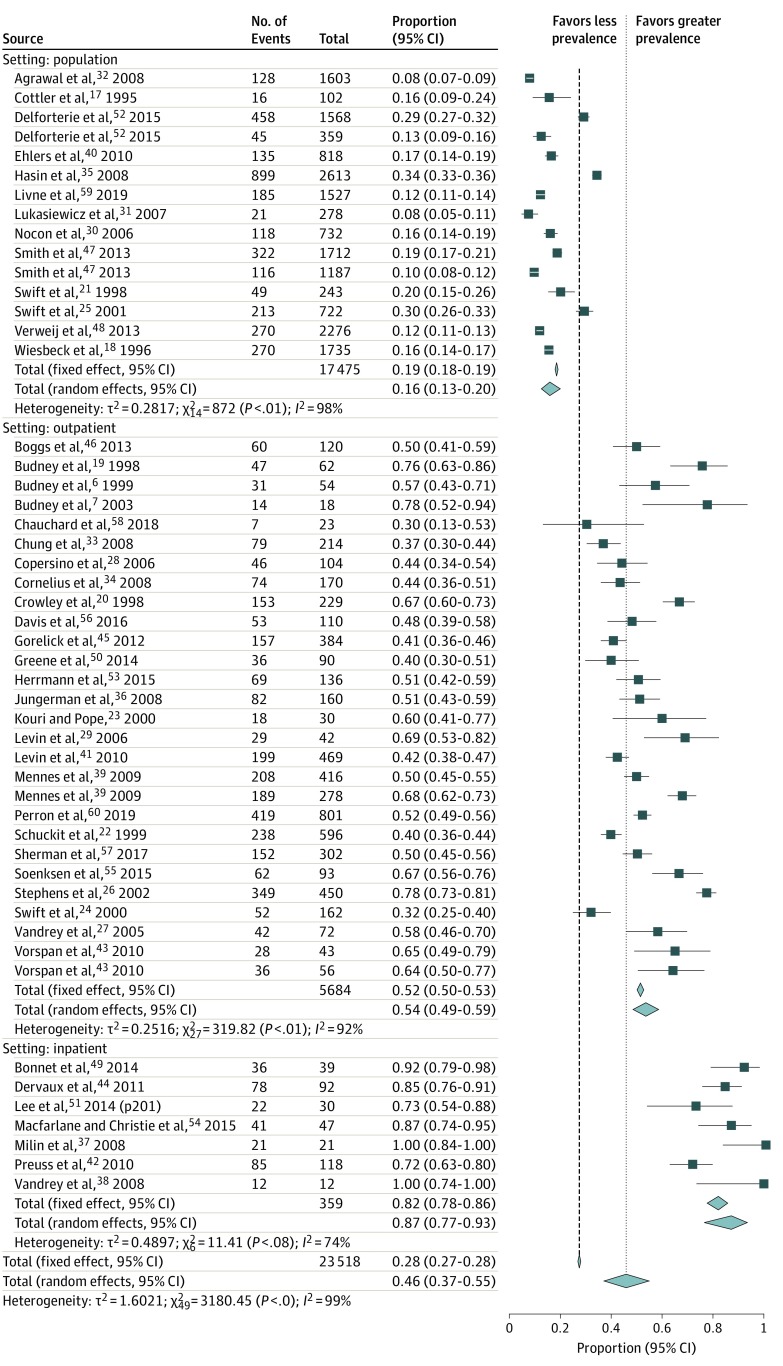
Prevalence of Cannabis Withdrawal in People With Cannabis Use Disorder Prevalence of cannabis withdrawal symptoms across 3 clinical settings: population-level samples, outpatient clinical samples, and inpatient clinical samples. The studies by Smith et al (2013), Mennes et al (2009), and Vorspan et al (2010) included 2 or more substudies.

Meta-analysis identified that the overall pooled prevalence of CWS in patients with regular or dependent use of cannabinoids was 47% (95% CI, 27%-37%). There was significant heterogeneity observed in this estimate (*I^2^* = 99.2%, *P* < .001; 50 studies; n = 23 518), with proportions of 0.16 (95% CI, 0.13-0.20) for population, 0.54 (95% CI, 0.49-0.59) for inpatient, and 0.87 (95% CI, 0.77-0.93) for inpatient (Figure 2). The range of CWS across studies varied from 8% to 100%.

When stratified by study setting, the prevalence of CWS in population-based samples was 17% (95% CI, 13%-21%; n = 15 studies; n = 17 475 participants), 54% in outpatient samples (95% CI, 48%-59%; n = 28 studies; n = 5684 participants), and 87% in inpatient samples (95% CI, 79%-94%; n = 7 studies; n = 357 participants). The difference between these groups was statistically significantly different (χ^2^ = 0.172, *P* < .001) (Table 2). Significant heterogeneity existed within the estimates for population-based samples (*I*^2^ = 98%), outpatient-based samples (*I*^2^ = 92%) and inpatient-based samples (*I*^2^ = 74%). The subgroup analysis based on sex did not find any differences in the prevalence of CWS among men (27%) compared with women (26%) (χ^2^ = 0.172, *P* = .99). Similarly, there was no association between CWS prevalence and age, race/ethnicity, method of CWS ascertainment, method of CUD diagnosis, comorbid alcohol use, comorbid psychiatric disorder, or geographic region. Furthermore, CWS estimates were significantly higher among studies measuring lifetime rather than current CWS prevalence (χ^2^ = 0.314, *P* < .001), among cohort studies rather than cross-sectional surveys (χ^2^ = 0.194, *P* < .001), and among studies involving participants who were seeking treatment compared with those who were not (χ^2^ = 446.32, *P* < .001).

**Table 2.** Subgroup Analyses of Factors Associated With Cannabis Withdrawal Syndrome Prevalence

Subgroup analyses	Prevalence (95% CI), %	Studies, No.	*z* Value	*I*^2^, %	*P* value	Between-group comparison	
						χ^2^	*P* value
Sample source^a^							
Population-based	17 (13-22)	15	7.866	98	<.001	0.172	<.001
Clinical							
Outpatient	54 (48-59)	28	20.267	94	<.001		
Inpatient	87 (79-94)	7	22.346	94	<.001		
Study design^a^							
Cross-sectional	19 (15.-24)	17	8.733	99	<.001	0.194	<.001
Cohort	62 (56-68)	33	19.816	96	<.001		
Method of CWS diagnosis							
Clinician-rated	52 (41-62)	20	9.725	99.	<.001	0.518	.49
Self-reported	45 (37-53)	23	11.086	99.	<.001		
Informant-rated	40 (21-59)	7	4.071	99	<.001		
Method of CUD diagnosis							
* DSM-IV*	49 (43-56)	37	14.993	99	<.001	0.493	.44
* DSM-III-R*	45 (31-58)	8	6.382	97	<.001		
* DSM-5*	34 (15-54)	5	3.470	99	<.001		
Timeline of CWS^a^							
Past year	31 (27-36)	32	13.590	99	<.001	0.314	<.001
Lifetime	76 (70-82)	18	26.659	86	<.001		
Sex							
Male	27 (21-34)	15	9.952	93	<.001	0.172	.99
Female	26 (19-33)	15	9.200	96	<.001		
Geographic region							
North America	48 (42-55)	38	14.637	99	<.001	0.484	.77
Europe	47 (25-70)	6	4.121	99	<.001		
South America	51 (44-59)	1	NA	NA	NA		
Australasia	36 (20-52)	5	5.385	99	<.001		

Abbreviations: CUD, cannabis use disorder; CWS, cannabis withdrawal syndrome; *DSM-III-R*, *Diagnostic and Statistical Manual, Third Edition-Revision; DSM-IV*, *Diagnostic and Statistical Manual, Fourth Edition*; *DSM-5*, *Diagnostic and Statistical Manual, Fifth Edition*; NA, not applicable.

^a^ Statistically significant at *P* < .05.

We used meta-regression to explore potential variables that may have accounted for the high heterogeneity observed for CWS prevalence (Table 3; eFigure 1 in the Supplement). Several methodologic features of studies and participant characteristics were significantly associated with CWS prevalence in meta-regression. The prevalence of CWS was higher with greater proportions of participants who reported daily cannabis use (β = 0.004, *P* < .001), had cannabis use disorder (β = 0.005, *P* < .001), had comorbid tobacco use (β = 0.002, *P* = .02), and had comorbid drug use (β = 0.003, *P* = .05).

**Table 3.** Meta-regression Analyses of Factors Associated With Prevalence of Cannabis Withdrawal

Meta-regression	β	Intercept	Studies, No.	*P* value
Disorder, %				
Alcohol use	−0.000	0.463	17	.97
Psychiatric	0.000	0.469	16	.93
Drug use	0.003	0.443	11	.05
Tobacco use	0.002	0.396	26	.02
Cannabis use	0.005	0.128	48	<.001
Mean age, y	−0.007	0.679	48	.10
Daily cannabis use, %	0.004	0.151	48	<.001
White race, %	0.001	0.433	42	.46

We explored the association of each study with pooled estimates via sensitivity analysis with leave-out-one meta-analysis, allowing the removal of each study from the evaluation. This analysis did not change the pooled prevalence of CWS substantially.

The potential for publication bias was assessed through funnel plots and by applying rank correlation tests, Egger tests, and the trim and fill method (eFigure 2 in the Supplement). The results did not suggest any evidence to support that a significant bias existed within this review.

Of the 50 studies, most (36 [72%]) had an overall rating of fair quality, while 2 studies (4%) were rated as good and 12 studies (24%) were rated as poor (eTable 3 in the Supplement). The most frequently met quality criteria were ascertainment of exposure, reported by 36 studies (72%), and comparability of cohorts on the basis of the design or analysis, reported by 26 studies (52%). A number of items were inconsistently completed, including demonstration that outcome of interest was not present at the start of the study, which was reported by 3 studies (6%), and adequacy of follow-up of cohorts, with rates of attrition and complete follow-up reported by 7 studies (14%).

## Discussion

Our systematic review and meta-analysis identified 50 studies that examined the prevalence of CWS. Overall, it was estimated that nearly half (47%) of all people with regular or dependent cannabinoid use will experience cannabis withdrawal. Other factors associated with CWS included study setting; concurrent tobacco, cannabis, and drug use disorders; and intensity of cannabis use. We did not find CWS to be associated with sex, age, race/ethnicity, or psychiatric comorbidity. The quality of the literature was rated as being fair for the majority of studies considered.

Many professionals and members of the general public may not be aware of cannabis withdrawal, potentially leading to confusion about the benefits of cannabis to treat or self-medicate symptoms of anxiety or depressive disorders.^80^ For example, when medical marijuana clients were asked about actual symptom relief, fewer than half report such relief,^81^ while others^82^ reported return of anxiety symptoms on cessation of use, suggesting the symptoms might be due to cannabis withdrawal.^83^ Because many CWS criteria are depression or anxiety symptoms, regular users may seek cannabis to obtain short-term symptom relief, unaware that this use could perpetuate a longer-term withdrawal problem.^77^

Clinicians should be aware of CWS as it is associated with clinically significant symptoms, which can trigger resumption of cannabis use and serve as negative reinforcement for relapse during a quit attempt.^28,41^ The clinical significance of CWS is shown by the fact that it can be impairing,^84^ that cannabis or other substances are used to relieve it, by its association with trouble quitting use,^28,41,85^ and by its negative prognostic association.^33,34,50,84^ The clinical significance of CWS is also supported by epidemiologic evidence, as studies involving latent variable modeling have shown that adding withdrawal to other CUD criteria improves model fit.^86^ Personality traits, psychiatric comorbidity, age at onset, level of cannabis use, severity of cannabis dependence, and concurrent drug and alcohol use have been proposed as other risk factors that may play a role in increasing risk of cannabis relapse following a quit attempt.^74^

When the *Diagnostic Manual of Mental Disorders, Fourth Edition*, was published, little was known about CWS, but in the ensuing 2 decades, substantial research efforts have advanced our understanding of CWS.^87,88,89^ Animal models have been helpful in elucidating the potential mechanisms and causes of CWS, with rodents exhibiting both tolerance and dependence following chronic administration of cannabinoids.^90^ Cannabis tolerance is known to be mediated by downregulation of the cannabinoid receptor type 1,^91^ which occurs more rapidly in cortical regions than in subcortical regions^92,93^ and is reversible on abstinence.^91^ Inhibitors of endocannabinoid-metabolizing enzymes reduce CWS responses among cannabis-dependent mice.^94^ Cannabis withdrawal syndrome and CUD are moderately heritable,^48^ implicating both genetic and environmental factors in their occurrence.

In our study, CWS was more frequently encountered among patients with comorbid tobacco and drug use. Although our study did not identify an association between psychiatric comorbidity or alcohol use and CWS prevalence, the prevalence of CUD comorbidity is known to be substantially higher among individuals with a primary anxiety,^44,95,96^ mood,^34,97^ eating,^61^ or psychotic disorder^46,98,99^ relative to the general population. These findings are consistent with comorbidity literature, which provides further support for the notion that the nature of associations between substance use and psychiatric disorders is usually adverse.^100^ As well, this association may be exacerbated by potential kindling effects induced by cannabis with the occurrence of other psychiatric conditions.^101^ An understanding of these risks may support clinicians in providing evidence-based care and appropriate counseling to their patients, particularly regarding cannabinoid stewardship.^101^

The finding that the prevalence of CWS was substantially higher in clinical populations—particularly inpatient samples—is consistent with the notion of a bidirectional association between cannabis use and mental health disorders.^102,103,104,105^ This finding is compatible with previous reviews, which have consistently reported that one-third of regular cannabis users in the general population^5,32,35^ and 50% to 95% of heavy users in treatment or research studies^28,33,34,41^ report symptoms of CWS. This finding may indicate that people with CWS are more likely to present to clinicians for help compared with those without CWS, notwithstanding the fact that CWS can be diagnosed and untreated.^10^ Whether there is an interaction or cumulative association between CWS prevalence and rates of presentation for clinical care is speculative at this point and requires further study. With this in mind, if CWS reflects underlying CUD pathologic factors, it may be an indication of underlying addictive burden and increase the likelihood of people being in clinical care as opposed to having CUD in the community without clinical support.^10^ The association between CWS and CUD may also be related to the central theories of substance initiation, whereby cannabinoids may be used to self-medicate psychiatric symptoms^106^ or may precipitate or aggravate existing mental health conditions.^107^

Several studies have attempted to determine the best tools for diagnosing CWS,^74,108^ but there has generally been poor correlation between rating scales. Despite within-sample heterogeneity, CWS prevalence estimates were similar irrespective of ascertainment method in our study. Stratification of CWS ascertainment methods did not reconcile heterogeneity in prevalence estimates; however, this does not mean that all CWS instruments are equal. Until there are methodologic guidelines and consensus on the best tools to screen for CWS, to our knowledge, these are the most comprehensive available data. The treatment of CUD is particularly challenging because there are no efficacious medications currently available, even with cannabinoid replacement therapies, such as nabilone, nabiximols, or dronabinol.^12,109^

### Strengths and Limitations

There are a number of strengths of this study. First, to our knowledge, this is the largest systematic review of cannabis withdrawal among people with CUD, and the first meta-analysis. Second, the quality of the majority of studies evaluated was fair. However, this study has limitations that should be considered in the appraisal of the evidence presented by this review. The largest limitation is the wide range of tools used to define CUD and CWS, which contributed to the large heterogeneity across studies. While the broad spectrum of included studies likely contributed to heterogeneity, the inclusion of only validated rating scales may have mitigated the heterogeneity somewhat. Although sex proportions were reported in overall samples, sex-specific prevalence estimates were only reported by a subset of studies (n = 15). As this is a study-level meta-analysis, a limitation of the methods is that individual-level characteristics were not explored. There was also limited representation of studies from all geographic regions, with only 1 study from South America and none from Africa; this limitation hampered our ability to estimate the prevalence of CWS across all continents. However, our subgroup analysis indicated that there was no significant difference in prevalence of CWS across the regions evaluated, which suggests that geographic regions may not play a substantial role in estimating CWS prevalence. There was also limited information about CWS in specific patient subgroups. There are other issues that are likely to influence CWS, which could not be addressed in this meta-analysis, including the changing products that are being used, which may affect tolerance, dependence, and CWS. However, this information is not available in most clinical studies to date. There was also a lack of individual-level analyses, which may be considered as another limitation of this study. In addition, few studies reported the amounts of concurrent substance use or cannabinoid levels in bodily fluids (eg, urine and blood), precluding a more focused analysis on the association between these measures and CWS prevalence.

## Conclusions

Cannabis withdrawal syndrome appears to be common among people with regular or dependent use of cannabinoids, with an overall pooled prevalence of 47% in this meta-analysis. Cannabis withdrawal syndrome was more common in men, participants from clinical samples, individuals with comorbid drug or tobacco use, and those with a higher level of cannabis use. Clinicians should be aware of the high prevalence of CWS and should consider screening for CWS, particularly among those who are at greater risk, in order to counsel patients and support individuals who are reducing their use of cannabis.

## Notes

Supplement. **eTable 1.** Full Systematic Review Search Strategy **eTable 2. **Study Characteristics **eTable 3. **List of Validated Cannabis Withdrawal Instruments and Scales **eFigure 1.** Subgroup Analyses and Meta-Regressions **eFigure 2.** Publication Bias Analysis Using Funnel Plot for Prevalence of Cannabis Withdrawal Syndrome Against Standard Error **eTable 3.** Quality Assessment Using Newcastle Ottawa Scale **eReferences**
