# Supplementary material for: Prevalence of Cannabis Withdrawal Symptoms Among People With Regular or Dependent Use of Cannabinoids: A Systematic Review and Meta-analysis
Source: JAMA Netw Open. 2020 Apr 9;3(4):e202370. doi: 10.1001/jamanetworkopen.2020.2370 (PMC7146100; doi:10.1001/jamanetworkopen.2020.2370)
Supplement: Supplement. — eTable 1. Full Systematic Review Search Strategy eTable 2. Study Characteristics eTable 3. List of Validated Cannabis Withdrawal Instruments and Scales eFigure 1. Subgroup Analyses and Meta-Regressions eFigure 2. Publication Bias Analysis Using Funnel Plot for Prevalence of Cannabis Withdrawal Syndrome Against Standard Error eTable 3. Quality Assessment Using Newcastle Ottawa Scale eReferences [file jamanetwopen-3-e202370-s001.pdf]

## Supplementary Online Content

Bahji A, Stephenson C, Tyo R, Hawken ER, Seitz DP. Prevalence of cannabis withdrawal symptoms among people with regular or dependent use of cannabinoids: a systematic review and meta-analysis. *JAMA Netw Open*. 2020;3(4):e202370. doi:10.1001/jamanetworkopen.2020.2370

**eTable 1.** Full Systematic Review Search Strategy

**eTable 2.** Study Characteristics

**eTable 3.** List of Validated Cannabis Withdrawal Instruments and Scales

**eFigure 1.** Subgroup Analyses and Meta-Regressions

**eFigure 2.** Publication Bias Analysis Using Funnel Plot for Prevalence of Cannabis Withdrawal Syndrome Against Standard Error

**eTable 3.** Quality Assessment Using Newcastle Ottawa Scale

**eReferences**

This supplementary material has been provided by the authors to give readers additional information about their work.

© 2020 Bahji A, et al. *JAMA Network Open.*

eTable 1. Full Systematic Review Search Strategy

***EMBASE: inception to June 15, 2019***

| Order | Search | Hits |
|---|---|---|
| 1. | exp "Cannabis (genus)"/ or cannabis.mp. or exp "cannabis use"/ or exp cannabis smoking/ or exp Cannabis sativa/ or exp "Cannabis sativa subsp. indica"/ or exp medical cannabis/ or exp cannabis addiction/ or exp cannabis/ or exp "Cannabis sativa subsp. sativa"/ or exp cannabis derivative/ | 47677 |
| 2. | marijuana.mp. | 16748 |
| 3. | 1 or 2 | 50463 |
| 4. | exp withdrawal syndrome/ or cannabis withdrawal.mp. | 34063 |
| 5. | exp drug withdrawal/ or exp withdrawal syndrome/ or withdrawal.mp. | 313158 |
| 6. | 4 or 5 | 313158 |
| 7. | prevalence.mp. or exp prevalence/ | 988826 |
| 8. | 3 and 6 and 7 | 239 |

***MEDLINE: inception to June 15, 2019***

| Order | Search | Hits |
|---|---|---|
| 1. | cannabis.mp. or exp Cannabis/ | 19938 |
| 2. | marijuana.mp. | 18422 |
| 3. | cannabinoid.mp. or exp Cannabinoids/ or exp Endocannabinoids/ | 24887 |
| 4. | hashish.mp. | 576 |
| 5. | exp "Marijuana Use"/ or exp Marijuana Abuse/ | 9816 |
| 6. | 1 or 2 or 3 or 4 or 5 | 47925 |
| 7. | withdrawal.mp. or exp Substance Withdrawal Syndrome/ | 94691 |
| 8. | cannabis withdrawal syndrome.mp. | 42 |
| 9. | 7 or 8 | 94691 |

© 2020 Bahji A, et al. *JAMA Network Open.*

| | | |
|---|---|---|
| 10 | exp Prevalence/ or prevalence.mp. | 654209 |
| 11 | 6 and 9 and 10 | 78 |

## *PsycINFO: inception to June 15, 2019*

| Order | Search | Hits |
|---|---|---|
| 1. | exp "Cannabis Use Disorder"/ or exp Cannabis/ or cannabis.mp. | 13157 |
| 2. | marijuana.mp. or exp Marijuana/ or exp Marijuana Usage/ | 11014 |
| 3. | cannabinoid.mp. or exp Cannabinoids/ | 5971 |
| 4. | exp Hashish/ or hashish.mp. | 397 |
| 5. | nabilone.mp. | 76 |
| 6. | exp Tetrahydrocannabinol/ or synthetic cannabinoids.mp. | 1659 |
| 7. | weed.mp. | 232 |
| 8. | 1 or 2 or 3 or 4 or 5 or 6 or 7 | 23957 |
| 9. | exp Drug Withdrawal/ or cannabis withdrawal.mp. | 7911 |
| 10 | withdrawal syndrome.mp. | 1637 |
| 11 | marijuana withdrawal.mp. | 93 |
| 12 | 9 or 10 or 11 | 8457 |
| 13 | 8 and 12 | 461 |

## *Web of Science: inception to June 15, 2019*

| Order | Search | Hits |
|---|---|---|
| 1. | TOPIC: (cannabis) | 21,176 |
| 2. | TOPIC: (withdrawal) | 103,958 |
| 3. | TOPIC: (prevalence) | 785,316 |
| 4. | 1 and 2 and 3 | 93 |

© 2020 Bahji A, et al. *JAMA Network Open.*

### *Allied and Complementary Medicine: inception to June 15, 2019*

| Order | Search | Hits |
|---|---|---|
| 1. | Cannabis/ or cannabis.mp. | 260 |
| 2. | marijuana.mp. | 92 |
| 3. | Cannabinoids/ or cannabinoids.mp. | 89 |
| 4. | 1 or 2 or 3 | 319 |
| 5. | withdrawal.mp. | 721 |
| 6. | cannabis withdrawal.mp. | 1 |
| 7. | 5 or 6 | 721 |
| 8. | 4 and 7 | 4 |

### *CINAHL and Pre-CINAHL: inception to June 15, 2019*

| Order | Search | Hits |
|---|---|---|
| 1. | (MH "Cannabis") OR "cannabis" OR (MH "Medical Marijuana") | 11,360 |
| 2. | (MH "Substance Withdrawal Syndrome") OR "withdrawal" | 16,343 |
| 3. | S1 AND S2 | 293 |

### *ProQuest Dissertations & Theses: inception to June 15, 2019*

| Order | Search | Hits |
|---|---|---|
| 1. | (cannabis withdrawal syndrome) AND prevalence | 3,094 |

### *Psychiatry Online: inception to June 15, 2019*

| Order | Search | Hits |
|---|---|---|
| 1. | cannabis withdrawal syndrome | 209 |

© 2020 Bahji A, et al. *JAMA Network Open.*

eTable 2. Study Characteristics

| Psych | TS | Demographic | CUD_% | Male_% | Age | White_% | Black_% | Hispanic_% | Asian_% | Daily_MJ_% | Timeline | N | CWS_n | CWS_% | Threshold_Sx | Quality |
|---|---|---|---|---|---|---|---|---|---|---|---|---|---|---|---|---|
| No | No | Adults | 12.2 | 62 | 30.8 | 76 | 0 | 19 | 7 | 100 | Past Year | 1603 | **128** | 8.0 | 2 | Fair |
| Yes | Yes | Adults | 81.7 | 77 | 41.5 | 31 | 69 | 0 | 0 | 53 | Past Year | 120 | **60** | 50.0 | 6 | Poor |
| Yes | Yes | Adults | 100 | 80 | 28.6 | 97 | 0 | | | 62 | Lifetime | 39 | **36** | 92.3 | 4 | Fair |
| No | Yes | Adults | 100 | 87 | 31.2 | 98 | 0 | 0 | 0 | 100 | Lifetime | 62 | **47** | 75.8 | 1 | Fair |
| No | Yes | Adults | 54 | 85 | 33.8 | 100 | 0 | 0 | 0 | 81 | Lifetime | 54 | **31** | 57.4 | 3 | Poor |
| No | Yes | Adults | 100 | 61 | 30.9 | 94 | 6 | 0 | 0 | 100 | Lifetime | 18 | **14** | 77.8 | 4 | Fair |
| Yes | No | Adults | 100 | 82.6 | 27.4 | 100 | 0 | 0 | 0 | 100 | Past Year | 23 | **7** | 30.4 | 3 | Poor |
| Yes | No | C&A | 60.7 | 67 | 16.8 | 90 | 5 | 2.5 | 2.5 | 45.3 | Past Year | 214 | **79** | 36.9 | 2 | Fair |
| No | No | Adults | 54 | 78 | 35 | 52 | 0 | 0 | 0 | 80 | Past Year | 104 | **46** | 44.2 | 4 | Poor |
| Yes | Yes | Mixed | 100 | 54 | 20.3 | 71 | 23 | | 1 | 49 | Past Year | 170 | **74** | 43.5 | 4 | Fair |
| No | No | Adults | 8 | 57 | 37 | 53 | 36 | 6 | 6 | 64 | Past Year | 102 | **16** | 15.7 | 3 | Fair |
| Yes | Yes | C&A | 78.6 | 72 | 15.8 | 48 | 12 | 41 | 0 | 71 | Lifetime | 229 | **153** | 66.8 | 1 | Fair |
| Yes | No | Mixed | 53.4 | 93 | 19.2 | 22 | 26 | 35 | 0 | 78 | Past Year | 110 | **53** | 48.2 | 1 | Fair |
| No | No | Adults | 11.7 | 50 | 24.8 | 100 | 0 | 0 | 0 | | Past Year | 1568 | **458** | 29.2 | 1 | Fair |
| No | No | Adults | 16.4 | 65 | 23.9 | 100 | 0 | 0 | 0 | 100 | Past Year | 359 | **45** | 12.5 | 1 | Fair |
| Yes | Yes | Adults | 100 | 75 | 28.7 | 100 | 0 | 0 | 0 | 100 | Lifetime | 92 | **78** | 84.8 | 1 | Fair |
| No | No | Adults | 13.9 | 38 | 48.4 | 29 | 71 | 0 | 0 | 100 | Past Year | 818 | **135** | 16.5 | 3 | Good |
| No | No | Adults | 92.4 | 58.3 | 29.2 | 0 | 82 | 6 | 0 | 66 | Past Year | 384 | **157** | 40.9 | 3 | Fair |
| Yes | No | C&A | 84.4 | 82 | 16.6 | 86 | 0 | 0 | 0 | 100 | Past Year | 90 | **36** | 40.0 | 1 | Fair |
| No | No | Adults | 57.2 | 67 | 58.5 | 75 | 11 | 7 | 2 | 57 | Past Year | 2613 | **899** | 34.4 | 3 | Fair |
| No | Yes | Adults | 77.9 | 73 | 33.3 | 10 | 85 | 5 | 0 | 100 | Past Year | 136 | **69** | 50.7 | 6 | Fair |
| No | Yes | Adults | 100 | 80 | 32.3 | 89 | 11 | 0 | 0 | 84 | Past Year | 160 | **82** | 51.3 | 4 | Fair |
| No | Yes | Adults | 100 | 87 | 42.5 | 85 | 15 | 0 | 0 | 100 | Lifetime | 30 | **18** | 60.0 | 3 | Good |
| No | Yes | Adults | 79.3 | 100 | 28.5 | 14 | 86 | 0 | 0 | 100 | Lifetime | 30 | **22** | 73.3 | 1 | Poor |
| Yes | Yes | Mixed | 100 | 74 | 34.3 | 42 | 24 | 29 | 2 | 42 | Lifetime | 42 | **29** | 69.0 | 1 | Fair |
| No | No | Adults | 91 | 58 | 31.2 | 20 | 80 | 0 | 0 | 100 | Past Year | 469 | **199** | 42.4 | 6 | Poor |

© 2020 Bahji A, et al. *JAMA Network Open.*

| | | | | | | | | | | | | | | | | |
|---|---|---|---|---|---|---|---|---|---|---|---|---|---|---|---|---|
| Yes | No | Adults | 24.6 | 66 | | 59 | | | | | Past Year | 1527 | **185** | 12.1 | 3 | Fair |
| Yes | No | Adults | 26.7 | 90.1 | 39 | | | | | 30.1 | Past Year | 278 | **21** | 7.6 | 3 | Fair |
| Yes | Yes | Adults | 100 | 63 | 31 | 66 | 0 | 0 | 34 | 98 | Lifetime | 47 | **41** | 87.2 | 1 | Poor |
| No | No | Adults | 48 | 49 | 22 | 100 | 0 | 0 | 0 | 100 | Past Year | 416 | **208** | 50.0 | 1 | Fair |
| No | Yes | Adults | 63 | 49 | 22 | 100 | 0 | 0 | 0 | 100 | Lifetime | 278 | **189** | 68.0 | 1 | Fair |
| Yes | Yes | C&A | 100 | 67 | 17 | 100 | 0 | 0 | 0 | 100 | Lifetime | 21 | **21** | 100.0 | 1 | Fair |
| No | No | Mixed | 3.5 | | 19 | 100 | 0 | 0 | 0 | 2.9 | Past Year | 732 | **118** | 16.1 | 4 | Fair |
| No | Yes | Adults | 1.8 | 53.6 | 45.1 | 86.7 | 10.7 | 8 | 8 | 87.2 | Past Year | 801 | **419** | 52.3 | 1 | Poor |
| No | Yes | Mixed | 100 | 85 | 19.6 | 100 | 0 | 0 | 0 | 100 | Lifetime | 118 | **85** | 72.0 | 3 | Fair |
| Yes | No | Adults | 30 | 66.1 | 32 | 76 | | | | | Past Year | 596 | **238** | 39.9 | 2 | Poor |
| No | Yes | Adults | 100 | 72 | 30.3 | 64 | 28 | 0 | 0 | 100 | Past Year | 302 | **152** | 50.3 | 1 | Fair |
| No | No | Adults | | 68 | 34.3 | | | | | 19 | Past Year | 1712 | **322** | 18.8 | 2 | Fair |
| No | No | Adults | | 68 | 34.3 | | | | | 19 | Past Year | 1187 | **116** | 9.8 | 2 | Fair |
| No | Yes | C&A | 76.9 | 100 | 16.4 | 45 | 22 | 27 | 5 | 51 | Lifetime | 93 | **62** | 66.7 | 1 | Fair |
| No | Yes | Adults | 100 | 68.4 | 36.1 | 69.3 | 12.2 | 17.3 | 1.1 | 100 | Lifetime | 450 | **349** | 77.6 | 1 | Fair |
| No | No | Adults | 57 | 58 | 36 | | | | | 65 | Past Year | 243 | **49** | 20.2 | 1 | Poor |
| No | No | Adults | 92 | 53.7 | 30 | | | | | 51.2 | Past Year | 162 | **52** | 32.1 | 1 | Fair |
| No | No | Adults | 20.8 | | | | | | | 100 | Past Year | 722 | **213** | 29.5 | 1 | Fair |
| No | Yes | C&A | 56.9 | 90 | 16.2 | 89 | 11 | 0 | 0 | 100 | Past Year | 72 | **42** | 58.3 | 4 | Fair |
| No | Yes | Adults | 100 | 50 | 28.2 | 100 | 0 | 0 | 0 | 100 | Lifetime | 12 | **12** | 100.0 | 1 | Fair |
| No | No | Adults | 23.6 | 39 | 31.9 | 100 | 0 | 0 | 0 | 14 | Past Year | 2276 | **270** | 11.9 | 3 | Fair |
| No | Yes | Adults | 79.1 | 69.8 | 37 | | | | | 100 | Lifetime | 43 | **28** | 65.1 | 2 | Poor |
| No | Yes | Adults | 100 | 71.4 | 27 | | | | | 100 | Lifetime | 56 | **36** | 64.3 | 2 | Poor |
| Yes | No | Adults | 50.4 | 63 | 32.3 | 72 | 19 | 6 | 0 | 16 | Past Year | 1735 | **270** | 15.6 | 2 | Fair |

© 2020 Bahji A, et al. *JAMA Network Open.*

**eTable 3.** List of Validated Cannabis Withdrawal Instruments and Scales

| Instrument | Type |
|---|---|
| Addiction Severity Index[1] | Clinician Administered |
| Cannabis Use Disorder and Associated Disabilities Schedule[2] | Clinician Administered |
| Customary Drinking and Drug Use Record[3] | Clinician Administered |
| Composite International Diagnostic Interview[4] | Clinician Administered |
| Circumstances, Motivation, Readiness for Treatment Scale[4] | Clinician Administered |
| Cannabis Use Disorders Identification Test[5] | Clinician Administered |
| Cannabis Withdrawal Scale[6] | Clinician Administered |
| Marijuana Problem Inventory[7] | Clinician Administered |
| Structured Clinical Interview for DSM[8] | Clinician Administered |
| Daily Marijuana Questionnaire[9,10] | Self-reported |
| Inventory of Drug Use Consequences[11] | Self-reported |
| Marijuana Craving Questionnaire[12] | Self-reported |
| Marijuana Quit Questionnaire[13] | Self-reported |
| Marijuana Motives Measure[14] | Self-reported |
| Marijuana Problem Scale[15] | Self-reported |
| Marijuana Withdrawal Symptom Checklist[16] | Self-reported |
| Symptom Checklist-90 revised[17] | Self-reported |
| Time-Line-Follow-Back[18] | Self-reported |

eFigure 1. Subgroup analyses and meta-regressions

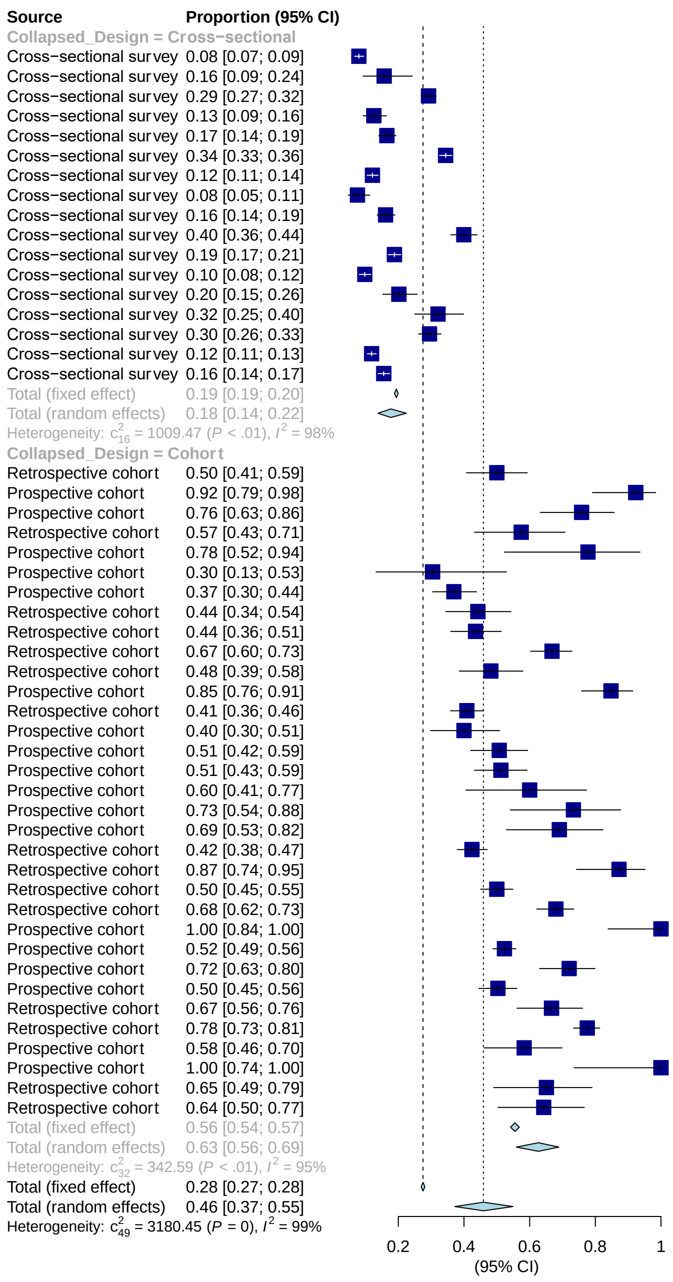


Forest Plot for Prevalence of Cannabis Withdrawal in People With Cannabis Use Disorder Stratified by Study Design

© 2020 Bahji A, et al. *JAMA Network Open.*

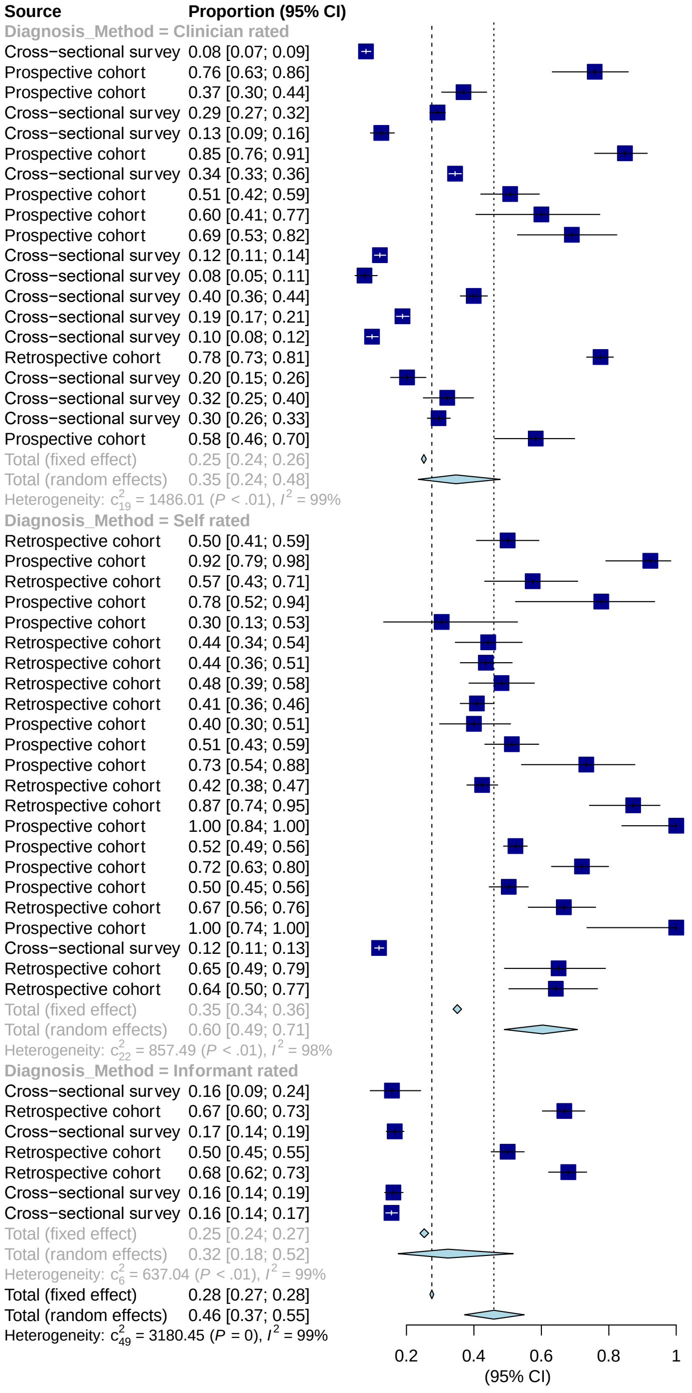


Forest Plot for Prevalence of Cannabis Withdrawal in People With Cannabis Use Disorder Stratified by Method of Cannabis Withdrawal Diagnosis

© 2020 Bahji A, et al. *JAMA Network Open.*

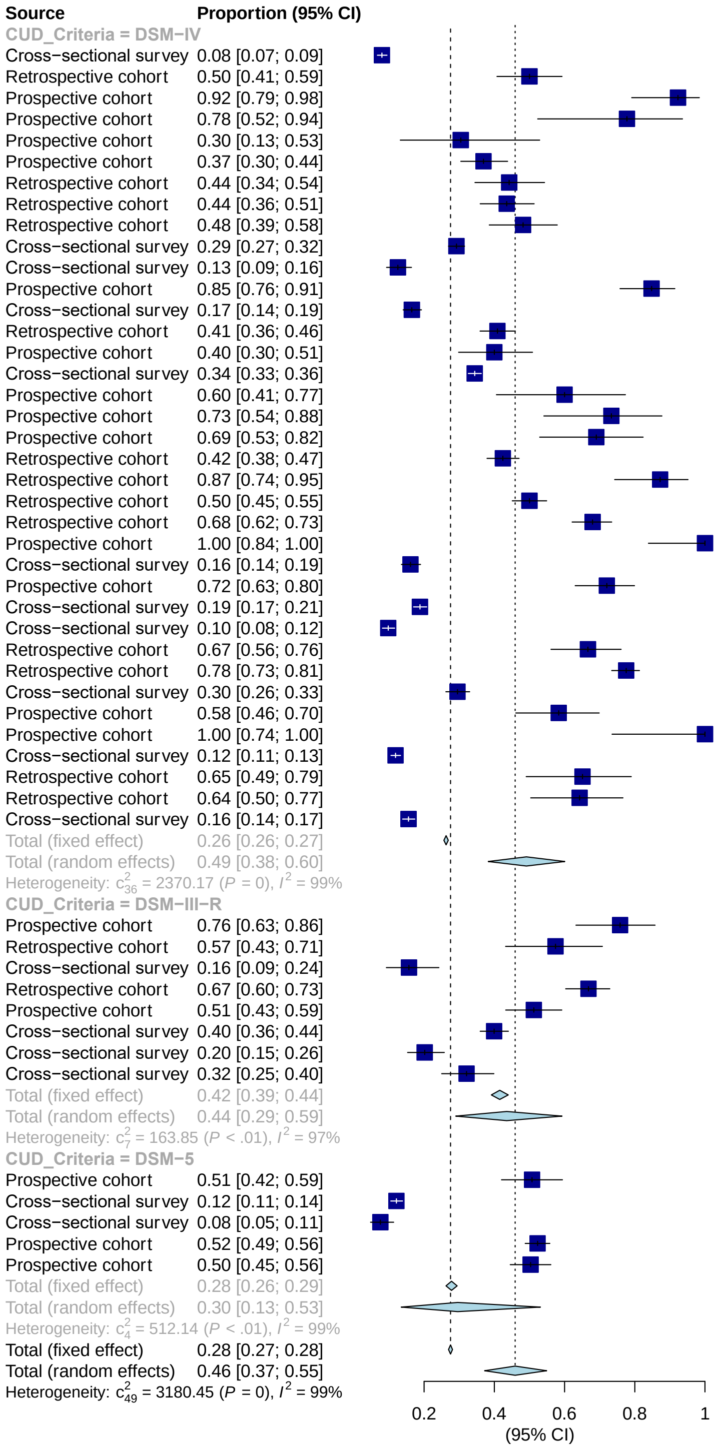


Forest Plot for Prevalence of Cannabis Withdrawal in People With Cannabis Use Disorder Stratified by Method of Cannabis Use Disorder Diagnosis

© 2020 Bahji A, et al. *JAMA Network Open.*

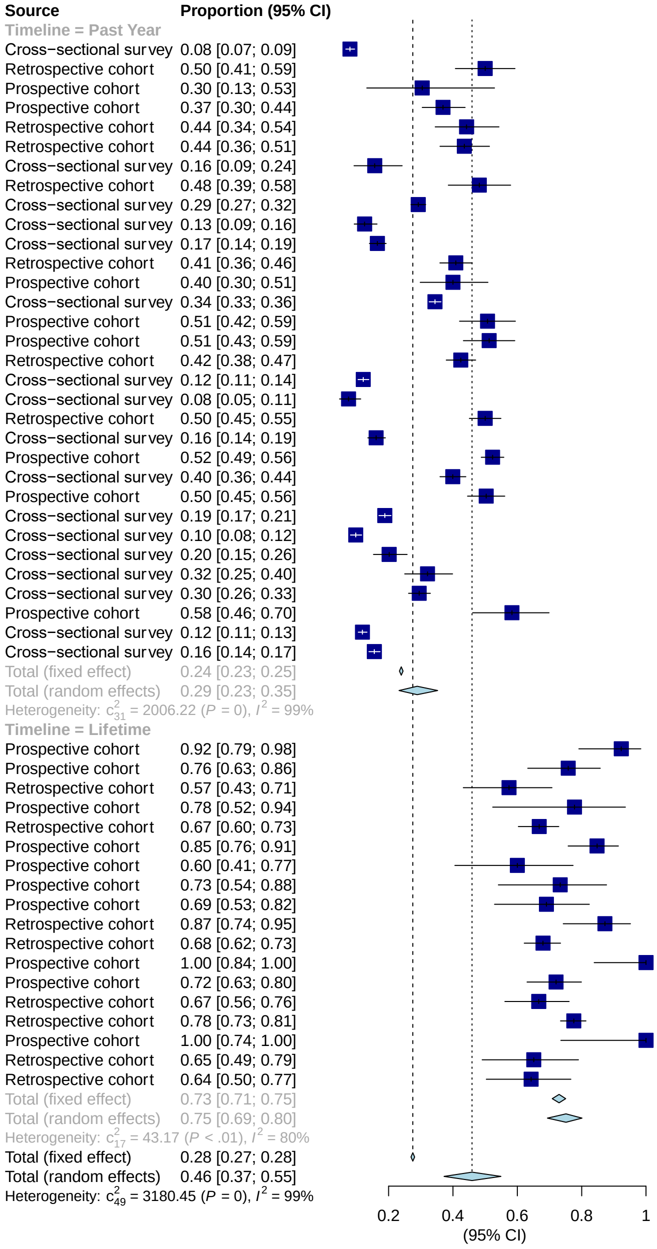


Forest Plot for Prevalence of Cannabis Withdrawal in People With Cannabis Use Disorder Stratified by Timeline of Cannabis Withdrawal

© 2020 Bahji A, et al. *JAMA Network Open.*

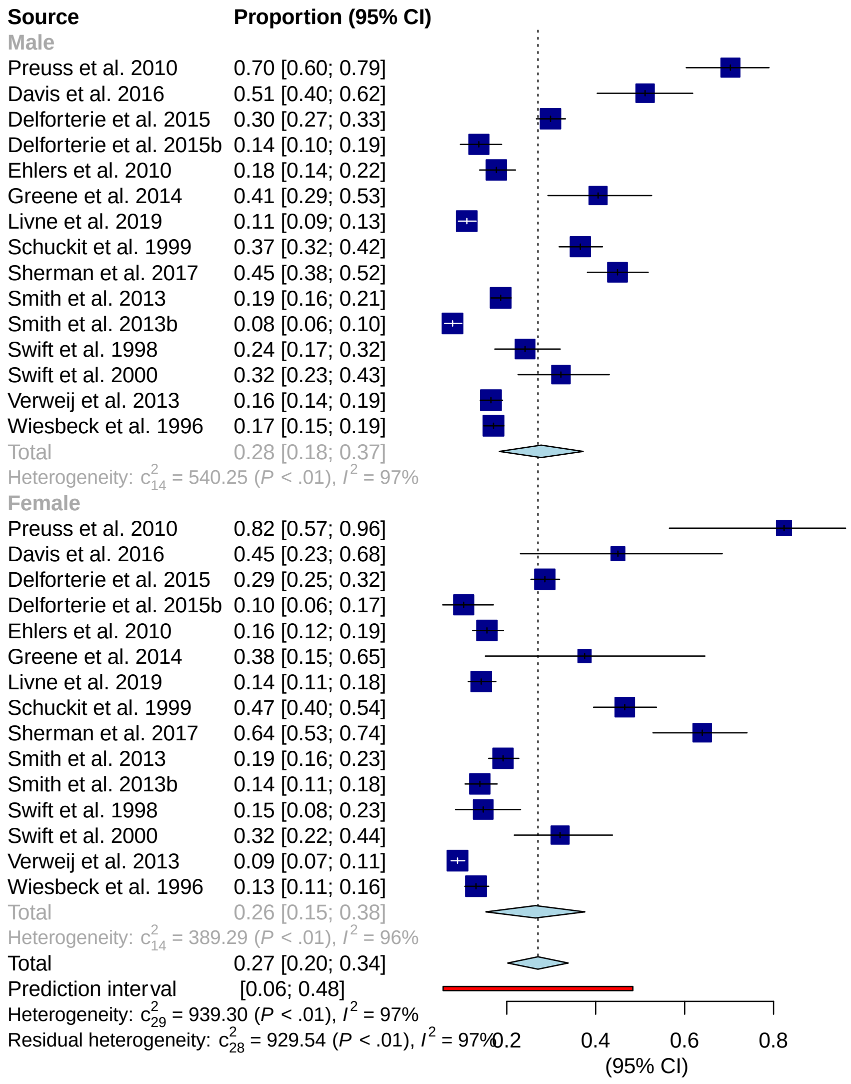


Forest Plot for Prevalence of Cannabis Withdrawal reported by studies with male and female subgroups

© 2020 Bahji A, et al. *JAMA Network Open.*

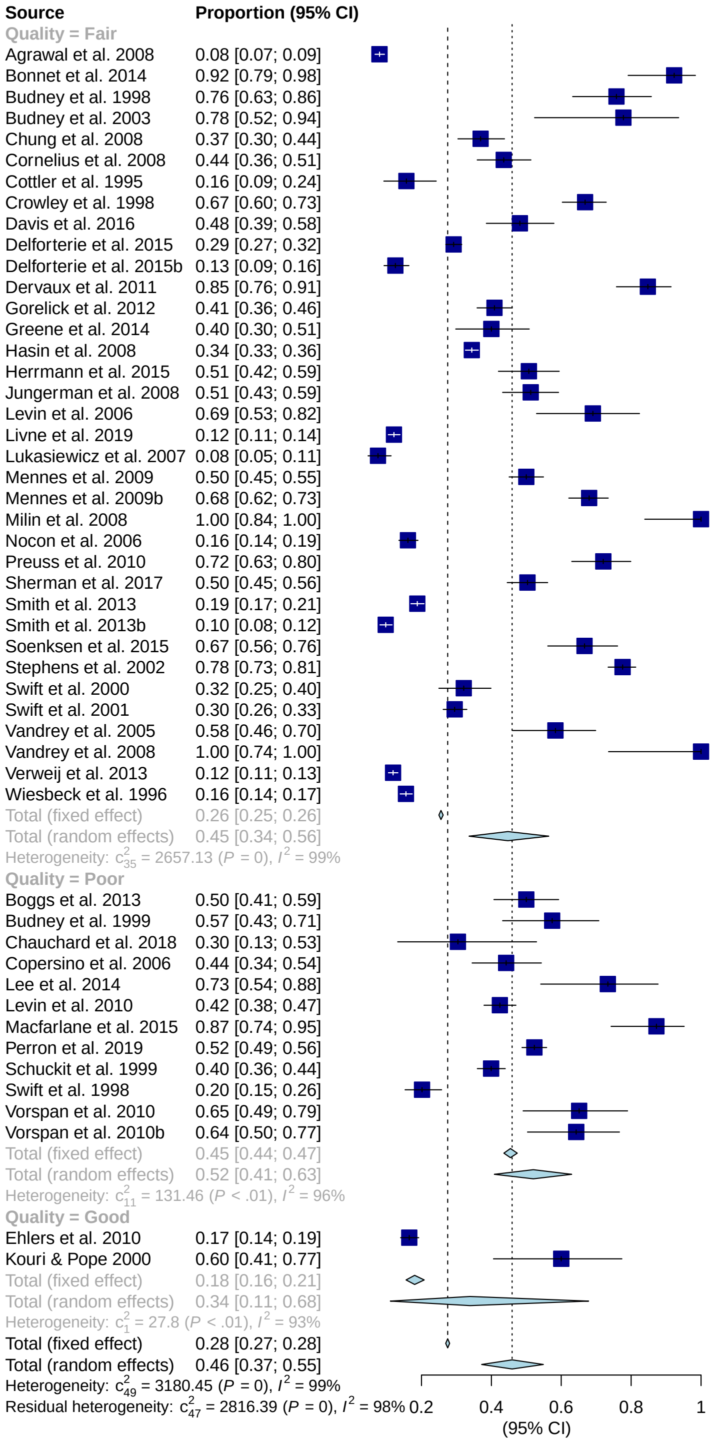


Forest Plot for Prevalence of Cannabis Withdrawal reported by studies by subgroups for quality of study (as per Newcastle Ottawa Scale): no subgroup differences detected.

© 2020 Bahji A, et al. *JAMA Network Open.*

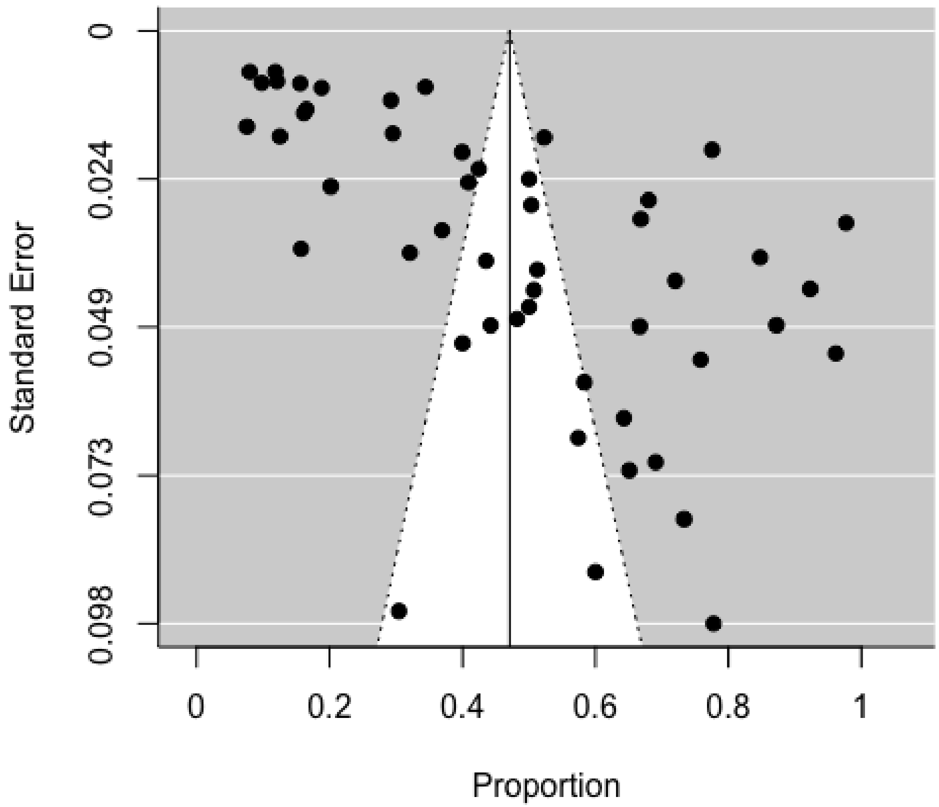


eFigure 2. Publication bias analysis using funnel plot for prevalence of cannabis withdrawal syndrome against standard error

© 2020 Bahji A, et al. *JAMA Network Open.*

eTable 3. Quality Assessment Using Newcastle Ottawa Scale

| ***Article*** | ***Representation*** | ***Selection*** | ***Exposure*** | ***Outcome*** | ***Comparability*** | ***Assessment*** | ***Follow-up*** | ***Attrition*** | ***Overall*** |
|---|---|---|---|---|---|---|---|---|---|
| ***Agrawal et al. 2008***[19] | * | * | * | | * | | | | *Fair* |
| ***Boggs et al. 2013***[20] | | | | | | | | | *Poor* |
| ***Bonnet et al. 2014***[21] | | | * | | | * | | * | *Fair* |
| ***Budney et al. 1998***[22] | | | * | | * | | | | *Fair* |
| ***Budney et al. 1999***[16] | | | | | | | | | *Poor* |
| ***Budney et al. 2003***[23] | | * | | | * | | * | | *Fair* |
| ***Chauchard et al. 2018***[24] | | | * | | | | | | *Poor* |
| ***Chung et al. 2008***[25] | | | * | | | | * | * | *Fair* |
| ***Copersino et al. 2006***[26] | | | * | | | | | | *Poor* |
| ***Cornelius et al. 2008***[27] | * | | * | | * | * | | | *Fair* |
| ***Cottler et al. 1995***[28] | * | | * | | | | | | *Fair* |
| ***Crowley et al. 1998***[29] | | | * | | * | | | | *Fair* |
| ***Davis et al. 2016***[30] | * | | * | | * | | * | | *Fair* |
| ***Delforterie et al. 2015***[31] | * | | * | | | | | | *Fair* |
| ***Delforterie et al. 2015b***[31] | * | | * | | | | | | *Fair* |
| ***Dervaux et al. 2011***[32] | | | * | | * | | | | *Fair* |
| ***Ehlers et al. 2010***[33] | * | * | * | | * | * | | * | *Good* |
| ***Gorelick et al. 2012***[34] | | | * | | * | | * | | *Fair* |
| ***Greene et al. 2014***[35] | | | | | * | * | | | *Fair* |
| ***Hasin et al. 2008***[36] | * | | * | | * | * | | | *Fair* |
| ***Herrmann et al. 2015***[37] | | | * | | * | | | | *Fair* |
| ***Jungerman et al. 2008***[38] | | | * | | * | | | | *Fair* |
| ***Kouri & Pope 2000***[39] | * | * | | * | * | | * | * | *Good* |
| ***Lee et al. 2014***[40] | | | | | * | | | | *Poor* |
| ***Levin et al. 2006***[41] | | | * | | * | | | | *Fair* |
| ***Levin et al. 2010***[13] | | | | | * | | | | *Poor* |
| ***Livne et al. 2019***[42] | * | | * | | | | | | *Fair* |

© 2020 Bahji A, et al. *JAMA Network Open.*

| | | | | | | | | | |
|---|---|---|---|---|---|---|---|---|---|
| **Lukasiewicz et al. 2007[43]** | | | * | | | * | | | *Fair* |
| **Macfarlane et al. 2015[44]** | | | * | | | | | | *Poor* |
| **Mennes et al. 2009[45]** | * | | * | | * | * | | | *Fair* |
| **Mennes et al. 2009b[45]** | * | | | | * | * | | | *Fair* |
| **Milin et al. 2008[46]** | | | * | | * | | * | | *Fair* |
| **Nocon et al. 2006[47]** | * | | * | | | | * | * | *Fair* |
| **Perron et al. 2019[48]** | | | | | | | | | *Poor* |
| **Preuss et al. 2010[49]** | | | | | * | | | * | *Fair* |
| **Schuckit et al. 1999[50]** | | | | | * | | | | *Poor* |
| **Sherman et al. 2017[51]** | | | * | | * | | | | *Fair* |
| **Smith et al. 2013[52]** | * | | * | | | * | | | *Fair* |
| **Smith et al. 2013b[52]** | * | | * | | | * | | | *Fair* |
| **Soenksen et al. 2015[53]** | | * | | * | * | | | | *Fair* |
| **Stephens et al. 2002[54]** | | * | * | | | * | * | | *Fair* |
| **Swift et al. 1998[55]** | | | * | | | | | | *Poor* |
| **Swift et al. 2000[56]** | | | * | | | | * | * | *Fair* |
| **Swift et al. 2001[57]** | * | | * | | | | | | *Fair* |
| **Vandrey et al. 2005[9]** | | | * | | * | | | | *Fair* |
| **Vandrey et al. 2008[58]** | | | * | * | * | | * | | *Fair* |
| **Verweij et al. 2013[59]** | * | | * | | | * | | | *Fair* |
| **Vorspan et al. 2010[60]** | | | | | | | | | *Poor* |
| **Vorspan et al. 2010b[60]** | | | | | | | | | *Poor* |
| **Wiesbeck et al. 1996[61]** | * | * | * | | | * | | | *Fair* |

*Studies that achieved a total rating of 6 points or higher were considered to be of the highest quality ("good"); studies that achieved a total rating of fewer than 2 points were considered to be of lowest quality ("poor"); and those between 2 and 5 points were rated as fair quality.*

© 2020 Bahji A, et al. *JAMA Network Open.*

## eReferences


1. McLellan AT, Kushner H, Metzger D, et al. The Fifth Edition of the Addiction Severity Index. J Subst Abuse Treat 1992;9(3):199–213.

2. Grant BF, Dawson DA, Stinson FS, et al. The Alcohol Use Disorder and Associated Disabilities Interview Schedule-IV (AUDADIS-IV): reliability of alcohol consumption, tobacco use, family history of depression and psychiatric diagnostic modules in a general population sample. Drug Alcohol Depend 2003;71(1):7–16.

3. Brown SA, Myers MG, Lippke L, et al. Psychometric evaluation of the Customary Drinking and Drug Use Record (CDDR): a measure of adolescent alcohol and drug involvement. J Stud Alcohol 1998;59(4):427–438.

4. Rubio-Stipec M, Bravo M, Canino G. [The Composite International Diagnostic Interview (CIDI): an epidemiologic instrument suitable for using in conjunction with different diagnostic systems in different cultures]. Acta Psiquiatr Psicol Am Lat 1991;37(3):191–204.

5. De Leon G, Melnick G, Kressel D, et al. Circumstances, motivation, readiness, and suitability (the CMRS scales): predicting retention in therapeutic community treatment. Am J Drug Alcohol Abuse 1994;20(4):495–515.

6. Allsop DJ, Norberg MM, Copeland J, et al. The Cannabis Withdrawal Scale development: patterns and predictors of cannabis withdrawal and distress. Drug Alcohol Depend 2011;119(1–2):123–129.

7. Knapp AA, Babbin SF, Budney AJ, et al. Psychometric assessment of the marijuana adolescent problem inventory. Addict Behav 2018;79113–119.

8. First MB. Structured Clinical Interview for the DSM (SCID). In: The Encyclopedia of Clinical Psychology. American Cancer Society; p1–6.

9. Vandrey R., Budney A.J., Kamon J.L., et al. Cannabis withdrawal in adolescent treatment seekers. Drug and Alcohol Dependence 2005;78(2):205–210.

10. Dimeff LA. Brief Alcohol Screening and Intervention for College Students (BASICS): A Harm Reduction Approach. Guilford Press; 1999. 218 p.

11. Tonigan JS, Miller WR. The inventory of drug use consequences (InDUC): test-retest stability and sensitivity to detect change. Psychol Addict Behav 2002;16(2):165–168.

12. Heishman SJ, Singleton EG, Liguori A. Marijuana Craving Questionnaire: development and initial validation of a self-report instrument. Addiction 2001;96(7):1023–1034.

13. Levin KH, Copersino ML, Heishman SJ, et al. Cannabis Withdrawal Symptoms in Non-Treatment-Seeking Adult Cannabis Smokers. Drug Alcohol Depend 2010;111(1–2):120–127.


© 2020 Bahji A, et al. *JAMA Network Open.*

14. Lee CM, Neighbors C, Hendershot CS, et al. Development and Preliminary Validation of a Comprehensive Marijuana Motives Questionnaire. J Stud Alcohol Drugs 2009;70(2):279–287.

15. Hodgins DC, Stea JN. Psychometric evaluation of a lifetime version of the marijuana problems scale. Addict Behav Rep 2018;821–24.

16. Budney AJ, Novy PL, Hughes JR. Marijuana withdrawal among adults seeking treatment for marijuana dependence. Addiction 1999;94(9):1311–1322.

17. Derogatis LR, Unger R. Symptom Checklist-90-Revised. In: The Corsini Encyclopedia of Psychology. American Cancer Society; p1–2.

18. Sobell LC, Sobell MB. Timeline Follow-Back. In: Litten RZ, Allen JP, editors. Measuring Alcohol Consumption: Psychosocial and Biochemical Methods. Totowa, NJ: Humana Press; p41–72.

19. Agrawal A, Pergadia ML, Lynskey MT. Is There Evidence for Symptoms of Cannabis Withdrawal in the National Epidemiologic Survey of Alcohol and Related Conditions? American Journal on Addictions 2008;17(3):199–208.

20. Boggs DL., Kelly DL., Liu F., et al. Cannabis withdrawal in chronic cannabis users with schizophrenia. Journal of psychiatric research 2013;47(2):240–5.

21. Bonnet U, Specka M, Stratmann U, et al. Abstinence phenomena of chronic cannabis-addicts prospectively monitored during controlled inpatient detoxification: Cannabis withdrawal syndrome and its correlation with delta-9-tetrahydrocannabinol and -metabolites in serum. Drug and Alcohol Dependence 2014;143(Complete):189–197.

22. Budney AJ, Radonovich KJ, Higgins ST, et al. Adults seeking treatment for marijuana dependence: a comparison with cocaine-dependent treatment seekers. Exp Clin Psychopharmacol 1998;6(4):419–426.

23. Budney AJ, Moore BA, Vandrey RG, et al. The time course and significance of cannabis withdrawal. J Abnorm Psychol 2003;112(3):393–402.

24. Chauchard E, Hartwell KJ, McRae-Clark AL, et al. Cannabis Withdrawal in Adults With Attention-Deficit/Hyperactivity Disorder. Prim Care Companion CNS Disord;20(1):. Epub ahead of print February 22, 2018. DOI: 10.4088/PCC.17m02203.

25. Chung T, Martin CS, Cornelius JR, et al. Cannabis withdrawal predicts severity of cannabis involvement at 1-year follow-up among treated adolescents. Addiction 2008;103(5):787–799.

26. Copersino M.L., Boyd S.J., Tashkin D.P., et al. Cannabis withdrawal among non-treatment-seeking adult cannabis users. American Journal on Addictions 2006;15(1):8–14.

© 2020 Bahji A, et al. *JAMA Network Open.*

27. Cornelius JR, Chung T, Martin C, et al. Cannabis withdrawal is common among treatment-seeking adolescents with cannabis dependence and major depression, and is associated with rapid relapse to dependence. Addict Behav 2008;33(11):1500–1505.

28. Cottler LB, Schuckit MA, Helzer JE, et al. The DSM-IV field trial for substance use disorders: major results. Drug and Alcohol Dependence 1995;38(1):59–69.

29. Crowley T.J., Macdonald M.J., Whitmore E.A., et al. Cannabis dependence, withdrawal, and reinforcing effects among adolescents with conduct symptoms and substance use disorders. Drug and Alcohol Dependence 1998;50(1):27–37.

30. Davis JP, Smith DC, Morphew JW, et al. Cannabis Withdrawal, Posttreatment Abstinence, and Days to First Cannabis Use Among Emerging Adults in Substance Use Treatment: A Prospective Study. J Drug Issues 2016;46(1):64–83.

31. Delforterie M., Creemers H., Agrawal A., et al. Functioning of cannabis abuse and dependence criteria across two different countries: the United States and The Netherlands. Substance use & misuse 2015;50(2):242–250.

32. Dervaux A., Krebs M.O., Laqueille X. Anxiety and depressive symptoms or disorders in patients with cannabis dependence without major psychiatric disorders. European Neuropsychopharmacology 2011;21(SUPPL. 3):S578–S579.

33. Ehlers CL., Gizer IR., Vieten C., et al. Cannabis dependence in the San Francisco Family Study: age of onset of use, DSM-IV symptoms, withdrawal, and heritability. Addictive behaviors 2010;35(2):102–10.

34. Gorelick D.A., Levin K.H., Copersino M.L., et al. Diagnostic criteria for cannabis withdrawal syndrome. Drug and Alcohol Dependence 2012;123(1–3):141–147.

35. Greene M.C., Kelly J.F. The prevalence of cannabis withdrawal and its influence on adolescents' treatment response and outcomes: A 12-month prospective investigation. Journal of Addiction Medicine 2014;8(5):359–367.

36. Hasin D.S., Keyes K.M., Alderson D., et al. Cannabis withdrawal in the United States: Results from NESARC. Journal of Clinical Psychiatry 2008;69(9):1354–1363.

37. Herrmann ES, Weerts EM, Vandrey R. Sex differences in cannabis withdrawal symptoms among treatment-seeking cannabis users. Exp Clin Psychopharmacol 2015;23(6):415–421.

38. Jungerman FS., Laranjeira R. Characteristics of cannabis users seeking treatment in São Paulo, Brazil. Revista panamericana de salud publica = Pan American journal of public health 2008;23(6):384–93.

39. Kouri EM, Pope HG. Abstinence symptoms during withdrawal from chronic marijuana use. Exp Clin Psychopharmacol 2000;8(4):483–492.

© 2020 Bahji A, et al. *JAMA Network Open.*

40. Lee D., Schroeder J.R., Karschner E.L., et al. Cannabis withdrawal in chronic, frequent cannabis smokers during sustained abstinence within a closed residential environment. American Journal on Addictions 2014;23(3):234–242.

41. Levin FR., Brooks DJ., Bisaga A., et al. Severity of dependence and motivation for treatment: comparison of marijuana- and cocaine-dependent treatment seekers. Journal of addictive diseases 2006;25(1):33–41.

42. Livne O, Shmulewitz D, Lev-Ran S, et al. DSM-5 cannabis withdrawal syndrome: Demographic and clinical correlates in U.S. adults. Drug and Alcohol Dependence 2019;195170–177.

43. Lukasiewicz M, Falissard B, Michel L, et al. Prevalence and factors associated with alcohol and drug-related disorders in prison: a French national study. Substance abuse treatment, prevention, and policy 2007;21.

44. Macfarlane V, Christie G. Synthetic cannabinoid withdrawal: A new demand on detoxification services. Drug and Alcohol Review 2015;34(2):147–153.

45. Mennes CE., Ben Abdallah A., Cottler LB. The reliability of self-reported cannabis abuse, dependence and withdrawal symptoms: multisite study of differences between general population and treatment groups. Addictive behaviors 2009;34(2):223–6.

46. Milin R, Manion I, Dare G, et al. Prospective Assessment of Cannabis Withdrawal in Adolescents With Cannabis Dependence: A Pilot Study. Journal of the American Academy of Child & Adolescent Psychiatry 2008;47(2):174–179.

47. Nocon A., Wittchen HU., Pfister H., et al. Dependence symptoms in young cannabis users? A prospective epidemiological study. Journal of psychiatric research 2006;40(5):394–403.

48. Perron BE, Holt KR, Yeagley E, et al. Mental health functioning and severity of cannabis withdrawal among medical cannabis users with chronic pain. Drug Alcohol Depend 2019;194401–409.

49. Preuss UW., Watzke AB., Zimmermann J., et al. Cannabis withdrawal severity and short-term course among cannabis-dependent adolescent and young adult inpatients. Drug and alcohol dependence 2010;106(2–3):133–41.

50. Schuckit MA, Daeppen J-B, Danko GP, et al. Clinical Implications for Four Drugs of the DSM-IV Distinction Between Substance Dependence With and Without a Physiological Component. AJP 1999;156(1):41–49.

51. Sherman BJ, McRae-Clark AL, Baker NL, et al. Gender differences among treatment-seeking adults with cannabis use disorder: Clinical profiles of women and men enrolled in the achieving cannabis cessation-evaluating N-acetylcysteine treatment (ACCENT) study. Am J Addict 2017;26(2):136–144.

© 2020 Bahji A, et al. *JAMA Network Open.*

52. Smith P.H., Homish G.G., Leonard K.E., et al. Marijuana withdrawal and aggression among a representative sample of U.S. marijuana users. Drug and Alcohol Dependence 2013;132(1–2):63–68.

53. Soenksen S, Stein LAR, Brown JD, et al. Cannabis Withdrawal Among Detained Adolescents: Exploring the Impact of Nicotine and Race. Journal of Child & Adolescent Substance Abuse 2015;24(2):119–124.

54. Stephens RS, Babor TF, Kadden R, et al. The Marijuana Treatment Project: rationale, design and participant characteristics. Addiction 2002;97(s1):109–124.

55. Swift W, Hall W, Didcott P, et al. Patterns and correlates of cannabis dependence among long-term users in an Australian rural area. Addiction 1998;93(8):1149–1160.

56. Swift W, Hall W, Copeland J. One year follow-up of cannabis dependence among long-term users in Sydney, Australia. Drug and Alcohol Dependence 2000;59(3):309–318.

57. Swift W., Hall W., Teesson M. Characteristics of DSM-IV and ICD-10 cannabis dependence among Australian adults: Results from the National Survey of Mental Health and Wellbeing. Drug and Alcohol Dependence 2001;63(2):147–153.

58. Vandrey RG, Budney AJ, Hughes JR, et al. A within-subject comparison of withdrawal symptoms during abstinence from cannabis, tobacco, and both substances. Drug and Alcohol Dependence 2008;92(1–3):48–54.

59. Verweij K.J., Agrawal A., Nat N.O., et al. A genetic perspective on the proposed inclusion of cannabis withdrawal in DSM-5. Psychological medicine 2013;43(8):1713–1722.

60. Vorspan F, Guillem E, Bloch V, et al. Self-reported sleep disturbances during cannabis withdrawal in cannabis-dependent outpatients with and without opioid dependence. Sleep Med 2010;11(5):499–500.

61. Wiesbeck G.A., Schuckit M.A., Kalmijn J.A., et al. An evaluation of the history of a marijuana withdrawal syndrome in a large population. Addiction 1996;91(10):1469–1478.

© 2020 Bahji A, et al. *JAMA Network Open.*
